# Incidence and Risk Factors of Spinal Anesthesia-Related Complications After an Elective Cesarean Section: A Retrospective Cohort Study

**DOI:** 10.7759/cureus.34198

**Published:** 2023-01-25

**Authors:** Reem A Algarni, Hadeer Y Albakri, Lamair A Albakri, Rawaf M Alsharif, Rawan K Alrajhi, Reham M Makki, Muhammad A Khan, Haifaa Kayal

**Affiliations:** 1 College of Medicine, King Saud Bin Abdulaziz University for Health Sciences, Jeddah, SAU; 2 College of Medicine, King Abdullah International Medical Research Center, Jeddah, SAU; 3 Department of Anesthesia, King Abdulaziz Medical City, Jeddah, SAU

**Keywords:** complications, cesarean section, bradycardia, hypotension, spinal anesthesia

## Abstract

Introduction

Neuraxial anesthetic techniques are the method of choice for cesarean section (CS) deliveries, and spinal anesthesia (SA) is the preferred technique. Although the use of SA has greatly improved the outcomes of CS deliveries, SA-related complications are still a matter of concern. The study's primary aim is to measure the incidence of SA complications after a CS, specifically hypotension, bradycardia, and prolonged recovery, as well as to identify the risk factors for these complications.

Method

The data of patients who had elective CS using SA from January 2019 to December 2020 was collected from a tertiary hospital in Jeddah, Saudi Arabia. The study design was a retrospective cohort study. The data collected included age, BMI, gestational age, comorbidities, the SA drug and dosage used, the site of the spinal puncture, and the patient's position during the spinal block. Also, the patient's blood pressure measurements, heart rate, and oxygen saturation levels were collected at baseline and at 5, 10, 15, and 20 minutes. SPSS was used for statistical analysis.

Results

The incidence of mild, moderate, and severe hypotension was 31.4%, 23.9%, and 30.1%, respectively. In addition, 15.1% of the patients experienced bradycardia, with 37.4% experiencing a prolonged recovery. Two factors were associated with hypotension, including BMI and the dosage of the SA, with a p-value of 0.008 and a p-value of 0.009, respectively. The site of the SA punctures equal to or lower than L2 was the only factor associated with bradycardia (p-value = 0.043).

Conclusion

The present study concludes that BMI and the dose of SA were the factors associated with SA-induced hypotension during a CS, and the site of the SA puncture equal to or lower than L2 was the only risk factor associated with spinal anesthesia-induced bradycardia.

## Introduction

Neuraxial anesthetic techniques are the method of choice for cesarean section (CS) deliveries. Spinal anesthesia (SA), in particular, is the preferred technique [1]. The use of neuraxial anesthesia is recommended to avoid the risks of aspiration or failed intubation associated with general anesthesia. It provides benefits that cannot be achieved with general anesthesia, including the immediate bonding of the mother and her neonate, the simplicity of the technique, the rapid onset of action, the reduced risk of systemic toxicity, and the prevention of pulmonary aspiration [2,3]. The use of neuraxial anesthesia in obstetrics resulted in significantly decreased anesthesia-related maternal mortality [4]. Although the use of SA has greatly improved the outcomes of CS, SA-related complications are still a matter of concern. According to a study, the most common complication caused by SA is SA-induced hypotension (SAIH) [1]. The incidence of SA-induced bradycardia is 2.5%, defined as a heart rate (HR) of less than 60 beats per minute [1]. Another well-known and frequently experienced complication is nausea and vomiting [5,6]. In addition to the physiological changes in pregnant women, a study by Fakherpour A et al. introduced several independent factors associated with SAIH in patients who had undergone an elective CS [7]. One of the factors is advanced age because older patients experience alterations in their sympathetic nervous system, baroreceptors, and cardiac reserve. Another risk factor for developing hypotension is a high BMI, which can cause elevated abdominal pressure, compression of the subarachnoid cavity, and a decrease in cerebrospinal fluid (CSF) volume, which increases the spinal blockade [7,8]. To improve the perioperative care of obstetric patients, an in-depth understanding of the topic of SA-related complications is essential. For this reason, this study aimed to measure the incidence of SA complications, focusing on hypotension, bradycardia, and prolonged recovery after elective CS. It also aimed to identify the factors associated with hypotension and bradycardia.

## Materials and methods

The research design was a retrospective cohort study conducted at King Abdulaziz Medical City (KAMC) in Jeddah, Saudi Arabia, from 2019 to 2020. The sample size was calculated using the Raosoft software, with a confidence level of 95%, a CI of 5%, and a population of 809. The target sample was calculated as 261 patients, estimated with a ±5% margin of error from the admitted obstetrics and gynecology patients at KAMC. A consecutive sampling technique was used. We included all females of child-bearing age who had undergone an elective CS under SA during the study period. The exclusion criteria were patients who received a combination of a spinal block with other types of anesthesia (an epidural block, inhalation or IV sedation, and general anesthesia); patients who received high doses of opioids (morphine >0.1 mg/kg; pethidine >1 mg/kg; fentanyl >1 microgram/kg) or sedative agents (midazolam >2 mg; ketamine >1 mg/kg; or propofol >1.5 mg/kg) within 60 minutes after the spinal block, patients who received a failed or partial SA; patients with chronic low back pain, diabetes, hypertension, coronary artery disease, a BMI of >40 kg/m2, a low sensory level, autonomic neuropathy, allergies to anesthetics, increased intracranial pressure, or discopathy; patients who were on selective serotonin reuptake inhibitors (SSRIs); patients whose surgery was longer than 120 minutes; and patients whose pregnancy was complicated by preeclampsia or eclampsia, multiple gestations, intrauterine growth restriction, severe congenital abnormalities, or stillbirth. A retrospective review of the records was performed, and a data collection sheet was used to gather the demographic data, including age, height, weight, and comorbid conditions. Data about the SA technique and conduct was obtained. Additionally, an intraoperative data section was used to record the intraoperative IV sedation medication used, the estimated blood loss, whether pre-hydration fluid and intraoperative fluids were administered, and if complications occurred during the operation.

SA-related complications include hypotension, categorized according to the degree of reduction from the baseline systolic blood pressure. For example, mild hypotension (decrease of ≥10% and ≤20%), moderate hypotension (decrease of >20% and ≤30%), and severe hypotension (decrease of >30%). In addition, bradycardia is defined as HR <60 beats per minute, and prolonged recovery is staying in the recovery room after the elective CS ≥45 minutes before transferring the patient to the regular ward). All the parameters gathered were coded into a Microsoft Office Excel program and then transferred to SPSS software version 20 (IBM Corp. Released 2011. IBM SPSS Statistics for Windows, Version 20.0. (IBM Corp., Armonk, NY, US) for statistical analysis. The quantitative variables, age, BMI, and gestational age, are described as 'median and IQR.' Line graphs were used for the graphical presentation of numerical data. The qualitative variables are summarized using appropriate descriptive statistics such as frequency and percentage. To compare the qualitative variables, a Chi-squared test or Fisher's exact test was used as required. A p-value <0.05 was considered significant. This study was approved by the institutional review board of King Abdullah International Medical Research Center.

## Results

Demographics

**Figure 1:**
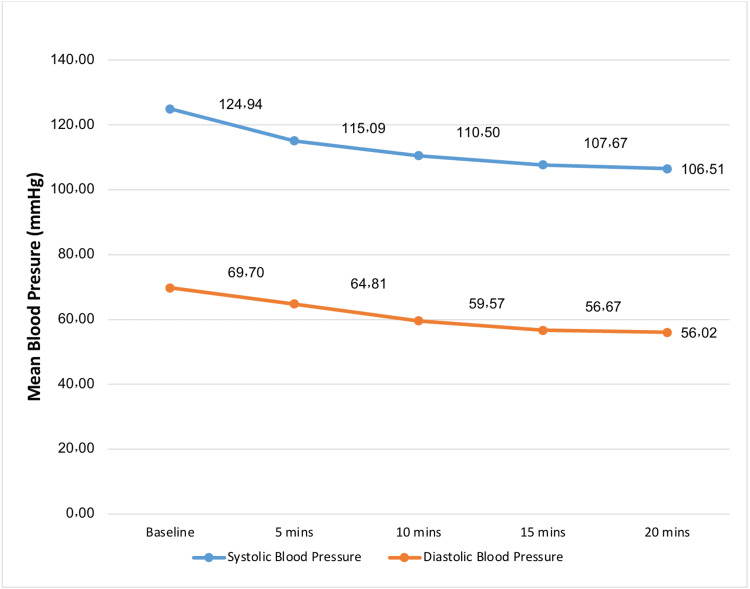
Mean blood pressure of patients who underwent cesarean section under spinal anesthesia.

**Figure 2:**
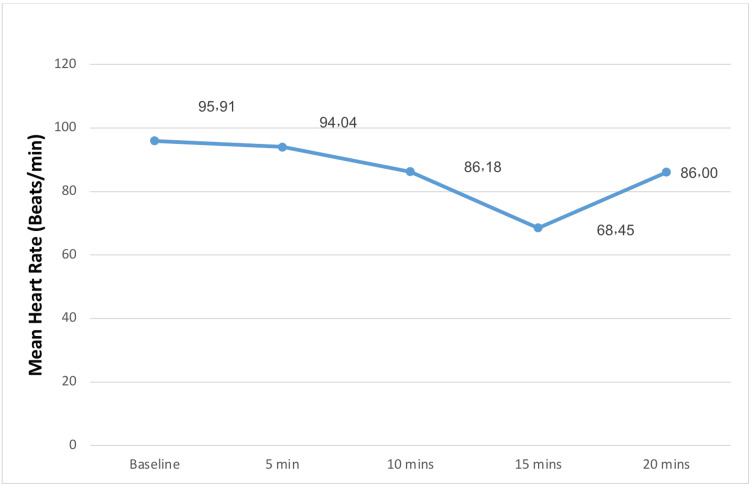
Mean heart rate of patients who underwent cesarean section under spinal anesthesia.

**Figure 3:**
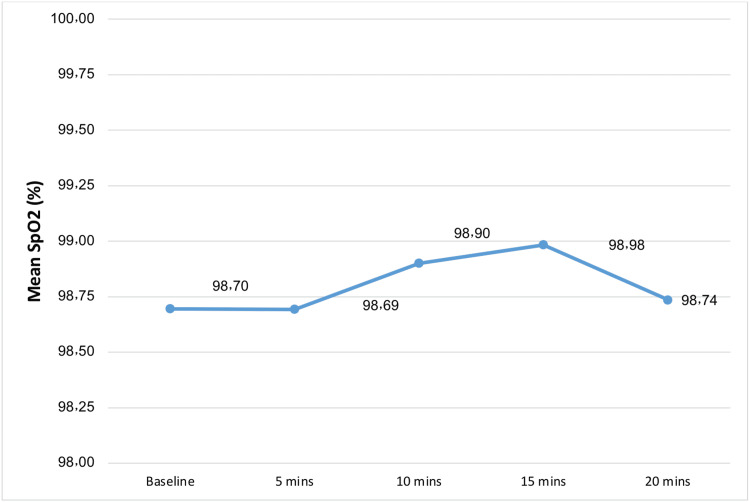
Mean oxygen saturation of patients who underwent cesarean section under spinal anesthesia.

The mothers' median age and gestational age were 32 years and 39 weeks, respectively, and the median BMI was 31.7 kg/m2. A description of mean maternal blood pressure, heart rate, and oxygen saturation is shown in Figures 1-3.

Preoperative and medical history

The baseline means of the SBP, HR, and SPO2 were 125, 77.5, and 98.9, respectively. A small proportion (n=36, 15.9%) had anemia, 21 patients (9.3%) had hypothyroidism, and 10 (4.4%) had respiratory disease. The majority (n=178) were gravida >2 (78.8%), 168 (74.3%) patients had two or more parities, and 40 patients (17.7%) had experienced a previous abortion. 

Anesthesia and drugs

Regarding the dose of anesthesia, 22 patients received less than 10 mg (9.8%), 176 patients received 10-12.5 mg (78.2%), and 27 patients more than 12.5 mg (12%). The site of SA puncture was at the level of L3-L4 in 109 patients (49.3%), L4-L5 in 83 patients (37.6%), and L2-L3 in 28 patients (12.7%). The majority (n=148, 84.1%) received SA in a lateral decubitus position, and 28 patients (15.9%) while sitting. 

Intraoperative variables

Preoperatively, 175 patients (77.4%) received preoperative fluid administration. Intraoperatively, only three of the patients (1.3%) received anticholinergic drugs, 77 patients (34.1%) received vasopressors, and 96 patients (51.3%) took antiemetic drugs. 

Incidence of SA-induced hypotension and complications

The incidence of mild, moderate, and severe hypotension was 31.4%, 23.9%, and 30.1%, respectively. In addition, 15.1% of patients had bradycardia, and 37.4% had a prolonged recovery. 

Associated factors

The findings revealed that two factors were associated with hypotension, including BMI and dose of SA, with a p-value of 0.008 and a p-value of 0.009, respectively (Table 1). The site of the spinal puncture equal to or lower than L2 was significantly associated with bradycardia with a p-value of 0.043 (Table 2).

**Table 1:** Factors associated with spinal anesthesia-induced hypotension.

Factors	Hypotension								P-value
	None		Mild		Moderate		Severe		
	n	%	n	%	n	%	n	%	
BMI (kg/m2)									
Underweight	1	50.0	0	0.0	0	0.0	1	50.0	0.008*
Normal	4	21.1	7	36.8	3	15.8	5	26.3	
Overweight	18	27.3	17	25.8	12	18.2	19	28.8	
Obese	10	7.2	47	33.8	39	28.1	43	30.9	
Age (year)									
< 30	12	17.1	17	24.3	16	22.9	25	35.7	0.122
30-39	18	13.8	50	38.5	28	21.5	34	26.2	
> 39	3	11.5	4	15.4	10	38.5	9	34.6	
Gestational age (week)									
34-38	10	10.6	37	39.4	18	19.1	29	30.9	0.089
> 38	23	17.4	34	25.8	36	27.3	39	29.5	
Smoking									
No	33	14.8	71	31.8	52	23.3	67	30.0	0.384
Yes	0	0.0	0	0.0	1	100.0	0	0.0	
Gravidity									
1	3	25.0	4	33.3	3	25.0	2	16.7	0.830
2	5	13.9	9	25.0	9	25.0	13	36.1	
> 2	25	14.0	58	32.6	42	23.6	53	29.8	
Parity									
0	3	23.1	5	38.5	3	23.1	2	15.4	0.395
1	10	22.2	10	22.2	11	24.4	14	31.1	
3	20	11.9	56	33.3	40	23.8	52	31.0	
Abortion									
0	28	15.1	63	33.9	40	21.5	55	29.6	0.542
1	3	11.5	5	19.2	9	34.6	9	34.6	
2 or more	2	14.3	3	21.4	5	35.7	4	28.6	
Spinal anesthesia drug									
Bupivacaine	1	8.3	3	25.0	2	16.7	6	50.0	0.599
Bupivacaine + Fentanyl	31	14.6	68	32.1	52	24.5	61	28.8	
Dose of anesthetics (mg)									
< 10	8	36.4	6	27.3	7	31.8	1	4.5	0.009*
10-12.5	22	12.5	59	33.5	39	22.2	56	31.8	
> 12.5	2	7.4	6	22.2	8	29.6	11	40.7	
Site of spinal puncture									
L2-L3	1	3.6	9	32.1	10	35.7	8	28.6	0.297
L3-L4	15	13.8	35	32.1	22	20.2	37	33.9	
L4-L5	16	19.3	26	31.3	21	25.3	20	24.1	
L5-S1	0	0.0	0	0.0	0	0.0	1	100.0	
Pre-hydration fluid administration									
No	9	17.6	18	35.3	9	17.6	15	29.4	0.611
Yes	24	13.7	53	30.3	45	25.7	53	30.3	
Intra-operative fluid volume administration									
No	5	23.8	6	28.6	4	19.0	6	28.6	0.654
Yes	28	13.7	64	31.4	50	24.5	62	30.4	
Use of anticholinergic agent									
No	33	14.9	70	31.7	53	24.0	65	29.4	0.470
Yes	0	0.0	0	0.0	1	33.3	2	66.7	
Use of vasopressors									
No	22	14.8	51	34.2	34	22.8	42	28.2	0.605
Yes	11	14.3	20	26.0	20	26.0	26	33.8	

**Table 2:** Factors associated with spinal anesthesia-induced bradycardia.

Factors	Bradycardia				P-value
	No		Yes		
	n	%	n	%	
BMI (kg/m2)					
Underweight	2	100.0	0	0.0	0.126
Normal	9	47.4	10	52.6	
Overweight	35	53.8	30	46.2	
Obese	91	65.9	47	34.1	
Age (year)					
<30	36	51.4	34	48.6	0.091
30-39	82	64.1	46	35.9	
>39	19	73.1	7	26.9	
Gestational age (week)					
34-38	61	66.3	31	33.7	0.187
>38	76	57.6	56	42.4	
Smoking					
No	135	61.1	86	38.9	0.392
Yes	0	0.0	1	100.0	
Gravidity					
1	7	58.3	5	41.7	0.978
2	22	61.1	14	38.9	
> 2	108	61.4	68	38.6	
Parity					
0	7	53.8	6	46.2	0.550
1	25	55.6	20	44.4	
3	105	63.3	61	36.7	
Abortion					
0	115	62.5	69	37.5	0.458
1	13	50.0	13	50.0	
2 or more	9	64.3	5	35.7	
Spinal anesthesia drug					
Bupivacaine	9	75.0	3	25.0	0.375
Bupivacaine + Fentanyl	127	60.2	84	39.8	
Dose of anesthetics (mg)					
< 10	15	71.4	6	28.6	0.587
10-12.5	105	60.0	70	40.0	
> 12.5	17	63.0	10	37.0	
Site of spinal puncture					
L2-L3	22	78.6	6	21.4	0.043*
L3-L4	68	63.0	40	37.0	
L4-L5	43	51.8	40	48.2	
L5-S1	1	100.0	0	0.0	
Pre-hydration fluid administration					
No	30	58.8	21	41.2	0.697
Yes	107	61.8	66	38.2	
Intra-operative fluid volume administration					
No	12	57.1	9	42.9	0.704
Yes	124	61.4	78	38.6	
Use of anticholinergic agents					
No	135	61.6	84	38.4	0.99
Yes	2	66.7	1	33.3	
Use of vasopressors					
No	88	59.9	59	40.1	0.582
Yes	49	63.6	28	36.4	

## Discussion

This retrospective study investigated the incidence of SA-associated complications in pregnant women who had elective C-sections. The incidence of mild, moderate, and severe hypotension was 31.4%, 23.9%, and 30.1%, respectively. Literature reported similar results that support our findings of a high incidence of hypotension related to the induction of SA during a C-section. In the Maayan-Metzger A et al. study, approximately half of the mothers experienced a decrease in their mean arterial blood pressure by ≥30% [9]. The Fakherpour A et al. prospective study reported the incidence of mild, moderate, and severe maternal hypotension as 20%, 35%, and 40%, respectively [7]. Varying incidence rates of hypotension induced by SA have been reported globally as 56.5%, 64%, and 79.6% [10,11,12]. However, other studies reported SAIH incidence rates ranging from 7% to 74%. This broad variation is attributed to discrepancies in the definition of hypotension used. A systematic review reported that 15 different definitions were used in the 63 studies included in the review [13]. The same review reported that the two most frequently used definitions were a decrease of <80% of the baseline value or the combination of SBP <100 mmHg and a decrease of <80% of the baseline [13]. Obstetric patients are more prone to SAIH due to various pathophysiological mechanisms. During pregnancy, sympathetic activity levels increase, and the sympathetic nerve fibers become more sensitive to anesthetics. A sympathetic nerve block by SA results in an increase in the parasympathetic tone of pregnant women compared to the parasympathetic response of the general population to SA [13]. The increased parasympathetic tone leads to systemic vasodilation, which reduces venous return to the heart. The reduced venous return is worsened by inferior vena cava (IVC) compression, leading to reduced cardiac return and output, eventually resulting in bradycardia, hypotension, nausea, and vomiting [13]. As a consequence of bradycardia and systemic hypotension, uterine and fetal blood flow may become compromised, resulting in fetal hypoxia and acidosis [5]. The present study showed that the risk of hypotension was significant in women with an increased BMI (p-value = 0.008). This finding agrees with Chinachoti T and Tritrakarn T study, which stated that BMI was a factor that increased the risk of hypotension [14]. This could be explained by the increased compression of the IVC, leading to hypotension [15]. More extension of a higher sympathetic blockade caused by compression of the subarachnoid space by the uterus and preganglionic sympathetic blockade might happen in obese pregnant women, which leads to the reduced sympathetic tone of the arterial circulation, vasodilatation of peripheral arteries and higher incidence of hypotension [16]. Moreover, as the intra-abdominal pressure increases during pregnancy, the CSF volume in the lumbar region will decrease, leading to hypotension [17]. Another of this study's findings associated with hypotension was the dose of anesthetics (p-value = 0.009). This finding is consistent with a study at Sriraj Hospital stating that a high dose of bupivacaine is a modifiable risk factor for SAIH [14]. Although our study showed no association between the SA puncture and hypotension level, another study indicated that the SA puncture level at L2-L3 is associated with hypotension [18].

Multiparous pregnant women with gravidity of ≥4 have a high risk of developing moderate to severe hypotension because of a significant decrease in peripheral vascular tone and vascular resistance [7]. In the current study, gravidity was not an identified risk factor for SAIH during CS. However, a study done at Chulalongkorn University, Bangkok, reported a significant correlation between the mother's age and the incidence of hypotension (p-value 0.030) [9]. The present study did not find an association between age and the risk of hypotension (p-value 0.122). This may be due to the good perioperative management of the women. In the present study, the incidence of bradycardia was 15.1%, which is similar to Chinachoti T and Tritrakarn T's study (16.4%) [14]. The site of SA puncture lower than or equal to L2 was significantly associated with developing bradycardia (p-value 0.043). However, no literature was reported investigating the association between bradycardia episodes and the site of the SA puncture. The main limitation of the current study is that the data were collected retrospectively from a single center in Jeddah. Thus, this limited sample may not represent the incidence of SA-induced complications in Saudi Arabia. The results shall be interpreted with caution when compared with other studies. In addition, because of the nature of this study as a retrospective study, some variables could not be controlled, like doses of local anesthetics and inconsistencies in the documentation of dermatome. Accordingly, we recommend future prospective studies to clearly distinguish between the mere association and causation among these variables and outcomes, namely hypotension and bradycardia, for better patient care and to prevent such significant complications. Also, we recommend modifications in the hospital anesthesia information system to help future researchers.

## Conclusions

To conclude, the incidence of mild, moderate, and severe hypotension after SA in our study was 31.4%, 23.9%, and 30.1%, respectively, and the incidence of bradycardia was 15.1%, similar to the literature. Research findings revealed that BMI and the dose of the SA were associated risk factors for hypotension associated with induction of SA during CS. However, the site of the SA puncture was the only identifiable risk factor associated with bradycardia. We advise healthcare practitioners and anesthetists to recognize and manage these risk factors to prevent SA-induced hypotension and bradycardia. Although more studies are needed to confirm the association between these findings, the risk factors identified could be a useful tool for decreasing the risk levels of SA complications.
